# Host species, pathogens and disease associated with divergent nasal microbial communities in tortoises

**DOI:** 10.1098/rsos.181068

**Published:** 2018-10-10

**Authors:** Chava L. Weitzman, Franziska C. Sandmeier, C. Richard Tracy

**Affiliations:** 1 Department of Biology, University of Nevada, Reno, NV 89557, USA; 2 Program in Ecology, Evolution, and Conservation Biology, University of Nevada, Reno, NV 89557, USA; 3 Department of Biology, Colorado State University—Pueblo, Pueblo, CO 81001, USA

**Keywords:** bacteria, *Gopherus*, microbiome, pyrosequencing, tortoise, upper respiratory tract disease

## Abstract

Diverse bacterial communities are found on every surface of macro-organisms, and they play important roles in maintaining normal physiological functions in their hosts. While the study of microbiomes has expanded with the influx of data enabled by recent technological advances, microbiome research in reptiles lags behind other organisms. We sequenced the nasal microbiomes in a sample of four North American tortoise species, and we found differing community compositions among tortoise species and sampling sites, with higher richness and diversity in Texas and Sonoran desert tortoises. Using these data, we investigated the prevalence and operational taxonomic unit (OTU) diversity of the potential pathogen *Pasteurella testudinis* and found it to be common, abundant and highly diverse. However, the presence of this bacterium was not associated with differences in bacterial community composition within host species. We also found that the presence of nasal discharge from tortoises at the time of sampling was associated with a decline in diversity and a change in microbiome composition, which we posit is due to the harsh epithelial environment associated with immune responses. Repeated sampling across seasons, and at different points of pathogen colonization, should contribute to our understanding of the causes and consequences of different bacterial communities in these long-lived hosts.

## 1. Introduction

Next-generation DNA sequencing technology has allowed the study of interactions among species in communities to extend beyond the focus of macro-organisms into the world of the microscopic. As in communities of macro-organisms, members of microbial communities participate in positive, negative and neutral interactions with other community members. Furthermore, microbial taxa interact with the larger organisms in, or on, which they reside. Many microbiome studies have been discovery-based, focusing on the identification of microbial species and their location on a host. Gut microbes and their roles in digestion and evolutionary transitions in diet have been especially well represented in the literature [1–7]. A recent review of studies of non-mammalian microbiomes found that most reptilian studies fail to address microbiomes other than those related to the gut [8].

Microbiomes may be particularly important for understanding infectious diseases. In disease ecology, characteristics of the host, its environment, pathogens and their interactive effects are synthesized to discuss the manifestation of disease. Recently, Hanson and Weinstock [9] proposed to add the microbiome as a fourth node to the disease ecology triangle, emphasizing the role of the microbial community in determining the way in which a pathogen harms its host. Epidemiological studies, particularly those involving human diseases [9], but also some wildlife diseases, consider the role of microbiomes increasingly more frequently [6,10–16]. One such disease garnering recent attention is chytridiomycosis, a pandemic affecting amphibian hosts caused by fungal pathogens. Amphibian skin harbours microbial communities that can inhibit pathogenic fungi [17,18]. Thus, the antimicrobial abilities of a host's microbiome could greatly impact whether an invading pathogen survives and thrives in the host. Conversely, invasion of amphibian skin by fungal pathogens alters the composition of skin microbial communities [19].

The microbiomes of amphibian skin have been at the forefront of emerging disease research; however, the microbiomes associated with other herpetofaunal diseases have been largely neglected thus far. One disease that could benefit from more microbial community research is an upper respiratory tract disease (URTD) in North American tortoise species. The tortoise genus *Gopherus* includes six species, four of which are located in the southern portion of the USA: Mojave desert tortoises (*G. agassizii*) in the southwest (California, Nevada, Utah, Arizona); Sonoran desert tortoises (*G. morafkai*) in the southwest (Arizona into Mexico); Texas tortoises (*G. berlandieri*) in Texas and into Mexico and gopher tortoises (*G. polyphemus*) in the southeast (Florida, Georgia, South Carolina, Alabama, Mississippi and Louisiana). In these species, multiple potential pathogens are associated with URTD, including *Mycoplasma agassizii*, *M. testudineum*, *Pasteurella testudinis*, an iridovirus and Testudinid herpesvirus 2 [20–25], and these pathogens may interact with the surrounding microbial community differently.

This respiratory disease describes signs including nasal exudate and lesions on the nasal epithelium, with extreme cases resulting in lethargy or even death [22]. Disease and transmission are thought to be pathogen load-dependent, and they are associated with increased levels of *M. agassizii* [26–28]. A closely related microbe, *M. testudineum*, is also associated with tortoise URTD, but its role in disease is less studied or understood [29–31]. It seems likely that pathogen proliferation in tortoise hosts occurs after an environmental stressor that has affected the host's immune system or caused a shift in microbial community interactions [32]. Importantly, this disease can be very slow to progress and take months to years from the time of pathogen invasion to the production of an adaptive immune response by the host and manifestation of clinical disease [33]. Clinical disease itself is a result of both host immune response and presence of the pathogen, and in URTD, inflammatory responses are associated with the slow induced antibody response of the host [22,33]. With a slow immune response, clinical signs of URTD can be present even when pathogens are not detected [34]. We currently know little about how microbial communities are affected by signs of clinical disease, both in the presence and absence of pathogens.

Studies of URTD have generally focused on detecting relevant pathogens, visually detecting signs of disease, and using serological tools to determine exposure to pathogens [35–40]. In Mojave desert tortoises, few studies have addressed the upper respiratory tract microbial community, and those that have, used labour-intensive culturing methods. Dickinson *et al*. [41] detected 19 taxa in nasal and cloacal samples from desert tortoises, while Ordorica *et al*. [42] found 260 bacterial isolates in the nasal passages of desert tortoises.

In the study reported here, we use next-generation DNA sequencing technology to describe the microbial communities in the upper respiratory tracts of four congeneric *Gopherus* tortoise species with the aim of detecting similarities and differences in communities among host species and sampling sites. We used these microbiome data to test two hypotheses regarding disease: (i) similar to microbial dynamics associated with other wildlife diseases such as chytridiomycosis, microbes associated with URTD are found in microbial communities with different compositions from those without pathogens; and (ii) the presence of nasal mucus correlates with differing microbial communities from those in tortoises without nasal mucus. Lastly, we use the microbiome data to evaluate the population diversity of *Pasteurella testudinis*, a microbe associated with the disease that has been the subject of little research.

## 2. Material and methods

### 2.1. DNA collection and microbiome sequencing

We sampled upper respiratory microbes from all four species of *Gopherus* found in the United States. A 3 ml nasal lavage (0.9% NaCl) [27,43] was collected from each tortoise using a sterile technique with sterile saline. Effluent lavage fluid was immediately collected back into the syringe to minimize exposure to air, and then the sample was preserved in RNAlater Stabilization Solution (Ambion Inc., Austin, Texas, USA) at a ratio of 1 : 5 preservative to sample volume. Samples were placed on ice in the field and frozen within 12 h of sampling. Gloves, syringes, needles (to extract the saline) and collection cups were discarded after working with each tortoise.

Samples represent three sites for each of the four *Gopherus* species, with two additional sites for Mojave desert tortoises (table 1). Tortoises were sampled from April 2010 to May 2015 (*G. agassizii* 2010–2012, *G. berlandieri* 2013–2014, *G. morafkai* 2012–2014, *G. polyphemus* 2013 and 2015).

**Table 1.** Sites sampled representing four North American *Gopherus* tortoise species, with sample size, average (±s.d.) number of sequences per sample after rare OTU filtering, number of samples with *Mycoplasma agassizii* and *M. testudineum* (adjusted sample size for missing data in parentheses), number of individuals with nasal mucus at the time of sampling and number of samples with *Pasteurella testudinis* (*Chelonobacter*).

	*n*	sequences	*M. agassizii*	*M. testudineum*	mucus	*P. testudinis*
*G. agassizii* (Mojave desert tortoises)						
Eldorado	11	2018 ± 1927	10	7	0	4
Fenner Valley	12	788 ± 1127	9	1	3	7
South Las Vegas	6	1065 ± 1110	4	0	3	4
Coyote Springs	9	1090 ± 772	1 (8)	0 (8)	0	5
Red Cliffs	5	492 ± 489	3	0	3	3
*G. morafkai* (Sonoran desert tortoises)						
Cave Buttes	7	2072 ± 2132	3	3	0	2
Silverbell	11	2262 ± 2862	0	0	0	7
Sugarloaf	13	2934 ± 2685	11	4	0	11
*G. berlandieri* (Texas tortoises)						
Chaparral WMA	13	1953 ± 2348	0	4	0	9
East Rio Grande	12	1084 ± 980	4	4	0	5
West Rio Grande	11	752 ± 340	1	2	0	7
*G. polyphemus* (gopher tortoises)						
Perdido	13	3851 ± 1371	5	0	2	12
Rayonier	11	2653 ± 1701	3	3	0	9
University of South Florida	12	2054 ± 1317	4	4	1	12

We extracted DNA from lavage samples with the Qiagen DNeasy Blood and Tissue Kit (Qiagen Inc., Valencia, CA, USA). Amplification of the variable regions V1–V3 of the bacterial 16S ribosomal RNA gene with the 28F/519R primer set (28F: 5′ GAGTTTGATCNTGGCTCAG 3′; 519R: 5′ GTNTTACNGCGGCKGCTG 3′) and subsequent pyrosequencing were conducted at the Research and Testing Laboratory (Lubbock, TX, USA). Bacterial tag-encoded FLX-Titanium (Roche, Nutley, NJ, USA) amplicon pyrosequencing (bTEFAP) followed the procedure described previously by Dowd *et al*. [44]. Pyrosequencing was multiplexed in four runs, and a list of the 8 bp barcodes used is available in the mapping file on Dryad. After sequencing, denoising and chimera checking were conducted at the Research and Testing Laboratory. Only sequences at least 225 bp in length and with an error-free barcode were included in downstream analyses.

When present, two pathogens of interest in this system, *Mycoplasma agassizii* and *M. testudineum*, are often detected in low abundance in nasal lavage samples. As such, these microbes are not likely to be found by pyrosequencing. Using methods described in Braun *et al*. [45], we conducted quantitative polymerase chain reaction (qPCR) to determine the presence or absence of *M. agassizii* and *M. testudineum* in our samples, which have been analysed elsewhere [31].

The following data processing and analyses were conducted using QIIME v. 1.9.1 [46], with default parameters unless otherwise stated. We used the split_libraries.py command to remove barcodes and demultiplex the data, with default quality filtering, which includes a quality score cut-off of 25 and a maximum of six ambiguous bases. We clustered operational taxonomic units (OTUs) using an open-reference OTU-picking process (pick_open_reference_otu.py), clustering sequences based on 97% similarity, and we identified taxonomy using the Greengenes database [47–49].

Only bacterial OTUs were kept, and OTUs were filtered to exclude those identified as mitochondrial and chloroplast (indicating plant origin). Before further filtering, the OTU table included 17 017 bacterial types. For analysis, we excluded OTUs present in only one sample and those that contained less than 0.01% of the total sequence abundance.

### 2.2. Bacterial communities among host species

In the programming language R v. 3.4.4 [50], we used ANOVA and Tukey's *post hoc* tests to detect differences in alpha diversity metrics (richness, Shannon diversity, Faith's phylogenetic diversity) among species and sites within species. After consideration of rarefaction curves (electronic supplementary material, figure S1), we rarefied data to 600 sequences per sample. We compared bacterial community composition among species and sites and based on the presence of *Mycoplasma* spp. with permutational analysis of variance (PERMANOVA) using the adonis function in the vegan package [51]. PERMANOVAs were conducted on both weighted and unweighted UniFrac distance matrices [52] with 999 permutations. Pairwise PERMANOVAs were conducted using the pairwise.perm.manova function in the RVAideMemoire package [53]. We used the Benjamini–Hochberg method [54] to adjust the *p*-value cut-off, decreasing the false discovery rate from PERMANOVAs. All PERMANOVA results in the study (including analyses presented below) were included in the *p*-value adjustment, allowing for a highest significant *p*-value of 0.027.

Using the pyrosequencing results, we sought to determine whether *Pasteurella testudinis*, a possible pathogen in *Gopherus* tortoises [21], was present in each sample. A study by Gregersen *et al*. [55] separated the genus *Chelonobacter* from other Pasteurellaceae, with *Chelonobacter* and *P. testudinis* forming a monophyletic group sister to other Pasteurellaceae including other *Pasteurella.* The reference database used in the present study, Greengenes [47,49] identified OTUs in our analyses as belonging in the *Chelonobacter* genus. However, another method of taxonomic identification, using USEARCH with the NCBI database, identifies these sequences as *P. testudinis*. Because sequence similarity between *P. testudinis* and *Chelonobacter* at the target sequence used by our pyrosequencing technique is unresolved, we analysed OTUs identified as *Chelonobacter* by Greengenes as a clade synonymous with the diverse strains of *P. testudinis* bacterium previously found in North American tortoises [21].

To compare communities with and without this microbe, the unrarefied data were filtered to exclude *Chelonobacter* OTUs, and we conducted principal coordinates analysis and PERMANOVAs within each tortoise species on rarefied 550 sequences per sample. Furthermore, we used pairwise PERMANOVAs between host species to compare the OTUs identified as being in the genus *Chelonobacter* by producing an OTU table containing only sequences from this genus.

We used co-occurrence analysis to determine which OTUs are associated with each host species and the presence or absence of *Chelonobacter*. With the CoNet application [56] in Cytoscape v. 3.6.1 [57], unrarefied OTU abundance data were normalized to account for sequencing depth, and taxa present in fewer than 10% of the samples were discarded. CoNet allows the user to implement multiple types of correlations and similarity/dissimilarity indices in analyses. We enabled Pearson and Spearman correlations, Bray–Curtis and Kullback–Leibler dissimilarities and mutual information (similarities) in our analyses, setting the threshold to include 1000 top and bottom scoring edges. We also enabled the program to compute correlations between higher-level taxa, i.e. bacterial taxon information above the level of OTU. Significant network connections between OTUs were determined using null distributions from 100 OTU-wise permutations and randomizations from 100 bootstrap distributions. The final analysis used Brown's method [58] to merge non-independent *p*-values and the Benjamini–Hochberg method [54] to adjust the *p*-value cut-off.

We conducted additional co-occurrence analyses, as above, to detect OTUs significantly associated with either *M. agassizii* or *M. testudineum* in Mojave desert tortoises, including OTUs in at least five samples in the analysis.

The core microbiome is the group of bacterial types present in most or all (depending on cut-off percentage) of the samples in a designated group. To detect the presence of prevalent bacterial taxa, we computed the core microbiome OTU composition, based on unrarefied data, for each tortoise species and each site separately.

### 2.3. Disease signs in Mojave desert tortoises

Because no Texas and Sonoran desert tortoises had nasal discharge at the time of sampling, and few gopher tortoises had discharge, we used data from three Mojave desert tortoise sampling locations (Fenner Valley, South Las Vegas and Red Cliffs; table 1) to detect differences in microbial communities in tortoises with and without visible mucus. Data from Coyote Springs and Eldorado were excluded in these analyses, because no individuals from those sites had nasal discharge at the time of sampling.

To determine whether the presence or absence of nasal discharge was associated with differing levels of OTU richness and diversity, we used ANCOVA with total sequences as a covariate. We used a covariate in place of rarefying the data to avoid excluding samples. Beta diversity, comparing diversity between samples, was evaluated with PERMANOVA and principal coordinates analysis on weighted and unweighted UniFrac distances. Diversity of *Chelonobacter* populations was also compared between samples from tortoises with and without nasal mucus using PERMANOVA.

We used co-occurrence analyses with these samples to determine which bacteria correlated with the presence or absence of nasal mucus. Only OTUs present in at least six samples were included in co-occurrence analyses.

## 3. Results

The final pyrosequencing dataset resulted in 7–9850 sequences per sample (median = 1093), totalling 869 bacterial OTUs. When data were rarefied to 600 sequences per sample, 37 samples were excluded from downstream analyses of alpha and beta diversity (Mojave desert tortoise, *n* = 18; Sonoran desert tortoise, *n* = 9; Texas tortoise, *n* = 7; gopher tortoise, *n* = 3).

### 3.1. Comparisons of bacterial communities among tortoise hosts

**Figure 1.**
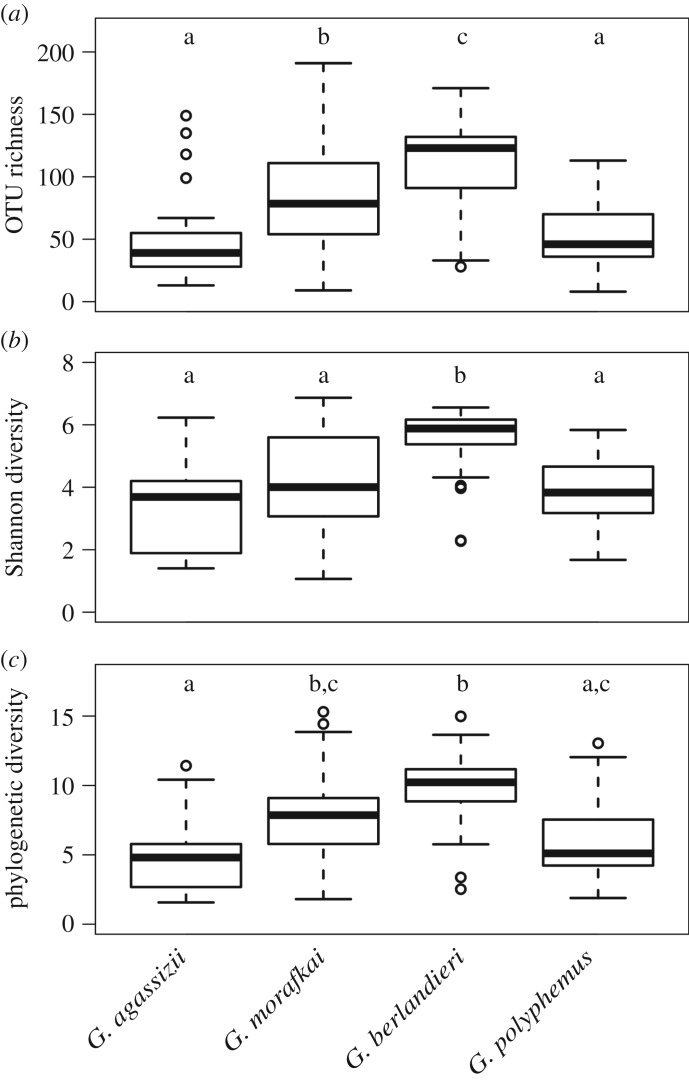
Alpha diversity metrics in North American *Gopherus* tortoise nasal samples. Data subset to 600 sequences per sample. (*a*) Observed OTU richness, (*b*) Shannon diversity and (*c*) Faith's phylogenetic diversity. Letters above the boxplots denote significantly different groups from Tukey's *post hoc* tests. Boxplots indicate the median, interquartile range, reasonable range of the data and outliers.

We detected differing bacterial richness among tortoise species (*F* = 18.98, *p* < 0.0001), with lower richness in Mojave desert tortoise and gopher tortoise samples and higher richness in Texas tortoise samples (figure 1*a*). Texas tortoise samples also had higher Shannon diversity (*F* = 13.72, *p* < 0.0001; figure 1*b*) and Faith's phylogenetic diversity (*F* = 15.72, *p* < 0.0001; figure 1*c*) than other species. Comparisons of richness among sites within each host species are presented in the electronic supplementary material, figure S3.

**Figure 2.**
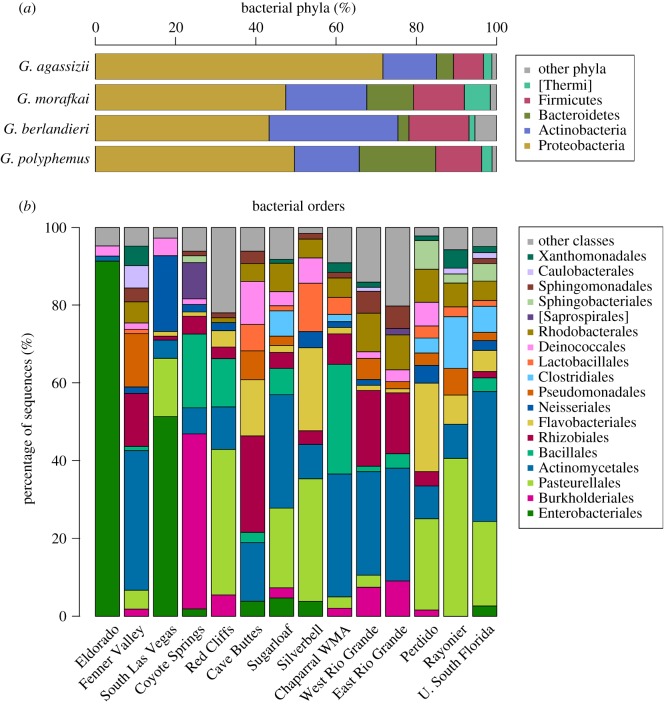
Proportion of bacterial taxa in samples rarefied to 600 sequences. (*a*) Bacterial phyla representing at least 5% of the sequences in any of the four *Gopherus* tortoise host species. (*b*) Bacterial orders representing at least 5% of the sequences in any one site. Data rarefied to 600 sequences per sample.

Bacteria in the phyla Proteobacteria, Actinobacteria, Bacteroidetes and Firmicutes collectively amounted to 92–97% of the rarefied sequences per tortoise species (figure 2*a*). Eighteen bacterial orders represented at least 5% of sequences for any one sampling location (figure 2*b*).

**Figure 3.**
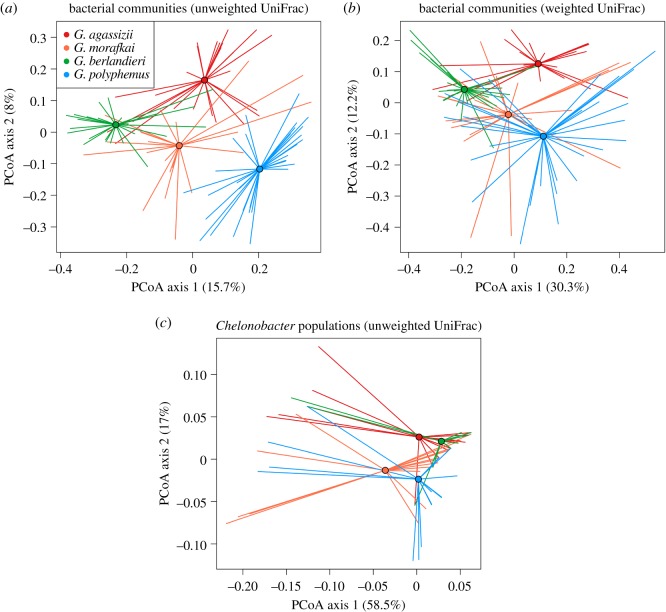
Principal coordinates analysis of nasal bacteria in four North American *Gopherus* tortoise host species. (*a*) Communities from 600 sequences per sample based on unweighted UniFrac distances. (*b*) Communities from 600 sequences per sample based on weighted UniFrac distances. (*c*) Diversity of *Chelonobacter* populations in tortoise host species. (*a–c*) Colour-coded by host species: red = *G. agassizii*; orange = *G. morafkai*; green = *G. berlandieri*; blue = *G. polyphemus*. Mean PCoA coordinates for each species are denoted by circles with vectors connecting means to individual points.

PERMANOVA detected significantly different communities among tortoise host species based on both weighted and unweighted UniFrac distances (*R*^2^ = 0.17, *p* = 0.001 each; figure 3*a*). All pairwise analyses were significant between species (*p* < 0.02 each). Sites sampled within tortoise species also hosted significantly different microbiota (*p* ≤ 0.003 each), though with weighted UniFrac distances, gopher and Sonoran desert tortoise communities did not differ among sampling sites. The few bacteria that were significantly associated with tortoise host species from co-occurrence analyses are presented in table 2. No OTUs were significantly associated with Mojave desert tortoises.

**Table 2.** Taxonomic identification of OTUs associated with tortoise species from co-occurrence analyses in CoNet. Each row represents one OTU.

host	phylum	class	order	family	genus
*G. morafkai*					
	Actinobacteria	Actinobacteria	Actinomycetales	Micrococcaceae	*Kocuria*
	[Thermi]	Deinococci	Deinococcales	Deinococcaceae	*Deinococcus*
*G. berlandieri*					
	Firmicutes	Bacilli	Bacillales	Staphylococcaceae	*Staphylococcus*
	Actinobacteria	Actinobacteria	Actinomycetales	Micrococcaceae	none
	Actinobacteria	Actinobacteria	Actinomycetales	Intrasporangiaceae	none
*G. polyphemus*					
	Proteobacteria	γ-Proteobacteria	Pasteurellales	Pasteurellaceae	*Chelonobacter*
	Bacteroidetes	Flavobacteriia	Flavobacteriales	[Weeksellaceae]	*Chryseobacterium*

The presence of *Mycoplasma agassizii* was correlated with different bacterial communities in Mojave desert tortoises (*R^2^* = 0.11–0.17, *p* < 0.008) and Sonoran desert tortoises (unweighted UniFrac, *R^2^* = 0.076, *p* < 0.027). Presence or absence of *M. testudineum* was also associated with differing microbiomes in Mojave desert tortoises (*R^2^* = 0.09–0.12, *p* ≤ 0.02). Co-occurrence analyses with Mojave desert tortoise data detected no OTUs significantly associated with the presence or absence of either *Mycoplasma* species.

To compare bacterial communities with and without *Pasteurella testudinis*, we assumed that OTUs identified as the genus *Chelonobacter* represented *P. testudinis* (see Material and methods) [55]. We excluded *Chelonobacter* sequences to compare bacterial communities with and without this microbe for each host species and rarefied the data to 550 sequences per sample. Bacterial communities differed between samples with and without *Chelonobacter* (*p* ≤ 0.022) when host species were pooled, but not within host species (*p* > 0.06 each). Co-occurrence analyses detected many co-presence associations between *Chelonobacter* OTUs. The absence of two *Chelonobacter* OTUs was also associated with the absence of *Ralstonia* (Betaproteobacteria: Burkholderiales: Oxalobacteraceae).

A total of 280 OTUs were identified as *Chelonobacter* across all host species, though only 14 of those were included in the OTU table after filtering out rare taxa (see Material and methods). *Chelonobacter* comprised up to 99% of the bacterial sequences in a sample (gopher tortoise from Rayonier, Alabama, including 32 OTUs) with up to 85 different *Chelonobacter* OTUs detected per sample (Sonoran desert tortoise from Sugarloaf, Arizona). Excluding rare OTUs, *Chelonobacter* populations were significantly different among all tortoise species (figure 3*c*; PERMANOVA, *R^2^* = 0.07–0.11, *p* ≤ 0.002). Gopher and Sonoran desert tortoise samples contained significantly different *Chelonobacter* types (unweighted) from each other and the other tortoise species (*p* ≤ 0.024 each). Gopher tortoises also differed from Texas and Mojave desert tortoises in *Chelonobacter* populations weighted for OTU abundance (*p* ≤ 0.002 each).

The purpose of a core microbiome analysis is to determine which OTUs are present in many or most individuals of a group, allowing us to detect potentially ecologically important taxa. The presence of a core microbiome varied widely by host species (electronic supplementary material, figure S4). In Mojave desert tortoise samples, zero OTUs were present in 50% of the samples, while 50% of Texas tortoise samples shared 41 OTUs, one of which (*Dietzia*) was found in 90% of the samples. Core microbiomes (50% cut-off) for gopher and Sonoran desert tortoises each included 30 OTUs (electronic supplementary material, figure S4). The number of OTUs found in 75% of samples from each site also varied (electronic supplementary material, figure S5), from zero in Red Cliffs samples (Mojave desert tortoises) to 26 in East Rio Grande samples (Texas tortoises; electronic supplementary material, figure S5). Four *Chelonobacter* OTUs were common in samples from gopher tortoises (all three sites) and Sonoran desert tortoises from Sugarloaf. Core microbiota (75% cut-off) from Sugarloaf, Perdido and University of South Florida shared three *Chelonobacter* OTUs, while Rayonier samples frequently had two of those common *Chelonobacter* OTUs plus an additional type not common in samples from any other site. Two *Chelonobacter* OTUs were found in all samples from the University of South Florida reserve (gopher tortoises).

### 3.2. Interaction with clinical signs of disease

**Figure 4.**
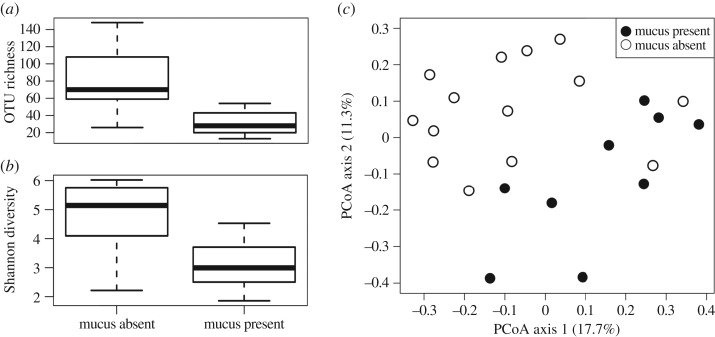
Effects of nasal mucus on upper respiratory bacteria in Mojave desert tortoises (*Gopherus agassizii*). Boxplots of (*a*) OTU richness (*F* = 16.26, *p* = 0.0007) and (*b*) Shannon diversity (*F* = 12.43, *p* = 0.002) with and without nasal discharge of mucus. Boxplots indicate the median, interquartile range and reasonable range of the data. (*c*) Principal coordinates analysis, with unweighted Unifrac distance matrix (*R^2^* = 0.09, *p* = 0.008) in tortoises with (closed circles) and without (open circles) nasal discharge of mucus.

In Mojave desert tortoises, the presence of exuded nasal mucus at the time of sampling was associated with a decrease in OTU richness (figure 4*a*; *F* = 16.26, *p* = 0.0007) and Shannon diversity (figure 4*b*; *F* = 12.43, *p* = 0.002) in lavage samples. Samples from tortoises with mucus also had significantly different bacterial community composition than those from tortoises without mucus (PERMANOVA, *R^2^* = 0.09, *p* = 0.008; figure 4*c*). This pattern was only observed with unrarefied data and with unweighted UniFrac distances, not when distances were weighted for OTU abundance. We analysed *Chelonobacter* populations between tortoises with and without secretions of nasal mucus and found no difference between the two groups (*R^2^* = 0.06, *p* > 0.5). Co-occurrence analyses did not detect any OTUs significantly associated, either positively or negatively, with the presence of nasal mucus.

## 4. Discussion

### 4.1. Tortoise upper respiratory microbiome

Using a next-generation DNA sequencing platform, we have determined the microbial diversity, community composition and core microbiome in the nares of four North American tortoise species in the context of a URTD and its associated bacterial pathogens. Our four tortoise species, and sites sampled within those species' ranges, had significantly different bacterial communities (figures 1 and 3; electronic supplementary material, figure S3). Accordingly, we did not find strong core microbiomes for each tortoise species, particularly for the two species of desert tortoises, and site-level core microbiomes varied in size across the sampling locations (electronic supplementary material, figure S5).

The spread of principal coordinates values from UniFrac distances (figure 3) suggests a high diversity of bacterial types capable of surviving in these varied tortoise host environments. Further research should address the degree to which microbial community composition or diversity is influenced by both abiotic and biotic environmental conditions for the host, such as rainfall, and transmission among tortoises. Moreover, we do not know if the patterns that we found are stable through time or space, and we know little about how nasal microflora change across seasons, with no studies addressing changes over the course of a host's lifetime. Using culturing techniques, Ordorica *et al*. [42] found changes in total bacteria count between seasons in Mojave desert tortoises, with higher counts in the summer versus autumn. We did not address annual or seasonal differences in nasal bacterial communities in this study, and we do not know how representative our findings are of the nasal bacterial communities tortoises interact with across their lifetimes.

The microbes surviving within the respiratory tracts of tortoises are probably a combination of some transmitted among tortoise hosts and some colonizing from the environment. The lack of a strong core microbiome in Mojave desert tortoises could mean that those hosts act more like distinct islands, with little opportunity for movement of bacteria among individuals. In these tortoises, microbiomes may function as isolated communities rather than as large contiguous metapopulations of associated bacteria.

Interestingly, the tortoise species most studied with regard to social structure and social interactions, the gopher tortoise [59], had a more widespread range of bacterial community composition visualized in principal coordinates space than Mojave desert tortoises or Texas tortoises. However, gopher tortoises did have a richer core microbiome than Mojave desert tortoises. We currently do not know if these differences are a function of social structure, host physiology, or host environment. Burrow sharing and other social interactions could increase the similarity in the upper respiratory microbiome, just as such activities may aid in the direct transmission of upper respiratory pathogens, by providing opportunities for direct transmission of bacteria between tortoises [26,60,61].

Because of the nasal lavage sampling protocol, some taxa found in this study could also be associated with the skin surrounding the nares, and we do not know if those bacteria are maintained in the tortoise respiratory tract. We predict that because reptile skin is not a mucous membrane, the skin microbiome would be more similar to the environment than that found in organisms such as amphibians. In sympatric amphibian species, cutaneous bacterial community composition is tightly linked to species identity [62,63]. If our upper respiratory tract data were strongly influenced by skin and environmental bacteria, then the additional bacteria should increase core microbiome membership, a result not supported in our data.

Similar to the skin microbiome in many other taxa [64], the majority of OTUs from the upper respiratory tracts of tortoises were in the bacterial phyla Proteobacteria, Actinobacteria, Firmicutes and Bacteroidetes. These phyla, plus Fusobacteria, are also commonly found in human upper respiratory tracts [65], with Bacteroidetes and Firmicutes dominating human lung bacteria [66]. Actinobacteria, however, comprised a greater percentage of Sonoran desert tortoise (20%) and Texas tortoise (32%) bacteria than that found on the skin of other non-human animals.

In North American tortoises, microbiome research involving next-generation sequencing technology has also been conducted on the faecal gut microbiome in gopher tortoises in Florida. Yuan *et al*. [5] discovered over 1000 core OTUs found in at least 90% of faecal samples. The large discrepancy between the presence of a strong core microbiome in the gut versus its absence in our nasal samples could be explained by the different roles played by the microbiome in different regions of the body, as well as different methods used to describe the host microbiome. Tortoises are herbivorous hind-gut fermenters, requiring a gut microbiome with large responsibility in the process of food digestion. The nasal flora, alternatively, probably play a role in disease avoidance [67]. While the gut microbiome is involved in both digestion and disease avoidance, the role of the respiratory tract microbiome in disease avoidance remains a relatively nascent field in immunology, especially in comparative immunology [16].

On epithelial tissue, microbes are important for mucosal homeostasis [68] and stimulating the immune system. Organisms with a healthy microbiome have greater immunocompetence than those with an altered or absent microbial community [69]. Importantly, microbes in any one mucosal location do not have effects limited to that location. For example, gut microbes can aid in fighting off respiratory pathogens [69]. As most research focuses on the importance of gut microbes, the importance of respiratory bacteria, particularly outside of the context of human disease, is still widely unknown (e.g. [70]).

### 4.2. Associations with pathogens and disease

Studies of URTD in North American tortoises have focused on *Mycoplasma* pathogens, with little research on the presence or effects of *Pasteurella testudinis* in the tortoise host. When *P. testudinis* was first described, multiple strains were found among different locations in the tortoise host, and this pathogen was described as being usually commensal with healthy tortoise hosts, though capable of causing disease [21]. Furthermore, within *P. testudinis* isolated from the upper respiratory tracts of Mojave desert tortoises, there is some differentiation between types more or less associated with URTD [71]. In our analyses using the Greengenes database, *P. testudinis* was not identified down to species but was placed within *Chelonobacter*, which may include multiple strains of *P. testudinis*, or multiple, related species.

The amount of diversity in our samples in the genus *Chelonobacter* was extremely large, though a previous study also found high ribotype diversity of *P. testudinis* in tortoises [71]. This bacterium and its genotypic diversity are geographically widespread in North American tortoises, but there are some differences in its populations among host species. Three *Chelonobacter* OTUs were common and shared among gopher tortoises and Sonoran tortoises from Sugarloaf. However, both Mojave desert tortoises and Texas tortoises had different *Chelonobacter* populations from those in gopher tortoises based on OTU identity and abundance, with additional differences in *Chelonobacter* populations when just considering OTU identity. The role of this microbe in the manifestation of URTD is still unclear. Mojave desert tortoises with and without nasal mucus did not have differing *Chelonobacter* populations, suggesting that if this microbe is associated with disease, then a bacterial strain alone is unlikely to be the cause of disease progression. Furthermore, the presence of *Chelonobacter* in the microbiome did not impact the remaining bacterial community composition within species, and it did not significantly co-occur with other microbes. Therefore, we did not find a clear indication that *Chelonobacter* is pathogenic or alters the microbiome in a negative way. We found similar results pertaining to the *Mycoplasma* pathogens associated with the disease.

In Mojave desert tortoises, the presence or absence of *Mycoplasma agassizii* and *M. testudineum* was associated with differing bacterial communities. This result suggests that different strains could be present in different tortoise host species, the pathogens' interactions with the different hosts affect the bacterial communities in differing ways, or some other aspect of the ecology of these *Mycoplasma* in the bacterial community differs among hosts. Where *M. agassizii* is associated with a shift in microbiome community, it is possible that tortoise hosts are colonized without disease-causing impacts until the community is pushed away from equilibrium by a stressor, with the shift in microbiome allowing *Mycoplasma* to switch from a commensal organism to one that causes URTD.

Our data on Mojave desert tortoises additionally found that nasal exudate, a clinical sign of URTD, was associated with a decrease in bacterial diversity (figure 4*a,b*) and a change in the bacterial community composition (figure 4*c*). Ordorica and colleagues [42], however, found that tortoises with clinical signs of URTD had larger and more diverse upper respiratory communities, opposite to our results. This may be due to differences in methodology, as they quantified cultured microbes. The changes in community structure in the presence of nasal mucus may allow for the growth of more easily cultured taxa. Many bacteria are not easily cultured, and thus the disparity between our results and those found by Ordorica *et al*. is not especially surprising. The nasal exudate we observed could also be a response to a pathogen that has not been tested for or some other cause of injury.

As a sign of disease, nasal exudate is associated with lesions, inflammation and sloughing off of mucosal and bacterial cells from the nasal epithelium [72]. This inflammatory response caused by the immune system influences the microbiota, possibly by forming an environment inhospitable to the maintenance of a healthy nasal microbiome. Immune responses to *Mycoplasma*, such as this inflammatory response, are frequently intermittent and ineffective at fully removing the pathogen, resulting in chronic infections [73]. This shift in the microbial community may also impact tortoises’ susceptibility to re-infection.

## 5. Conclusion

Tortoise upper respiratory tracts harbour thousands of bacterial types, forming diverse community assemblages. The presence of pathogens and disease may alter the natural microbiome in tortoise hosts, depending on both the pathogen and host in question. Importantly, clinical signs of disease, associated with inflammation and sloughing off of cells, dramatically alter the upper respiratory microbiome, and long-term effects of microbial shifts are yet unknown. The tortoise upper respiratory microbiome is diverse and probably important for normal host physiology; the roles of tortoise social interactions, wet and dry seasons and ontogenetic changes in the maintenance and stability of the microbiome are yet to be addressed.

## Supplementary Material

Supplementary Figures

## Data accessibility

The biom table resulting from open-reference OTU picking on our 454-pyrosequencing and an associated sample data file have been uploaded on Dryad: http://dx.doi.org/10.5061/dryad.7f2v776 [74].
