# Supplementary material for: Host species, pathogens and disease associated with divergent nasal microbial communities in tortoises
Source: R Soc Open Sci. 2018 Oct 10;5(10):181068. doi: 10.1098/rsos.181068 (PMC6227988; doi:10.1098/rsos.181068)

# Weitzman, Sandmeier, & Tracy. Host species, pathogens, and disease associated with divergent nasal microbial communities in tortoises. Royal Society open science.

Supplemental Figures

Figure S1. Rarefaction curves of OTU richness by species.


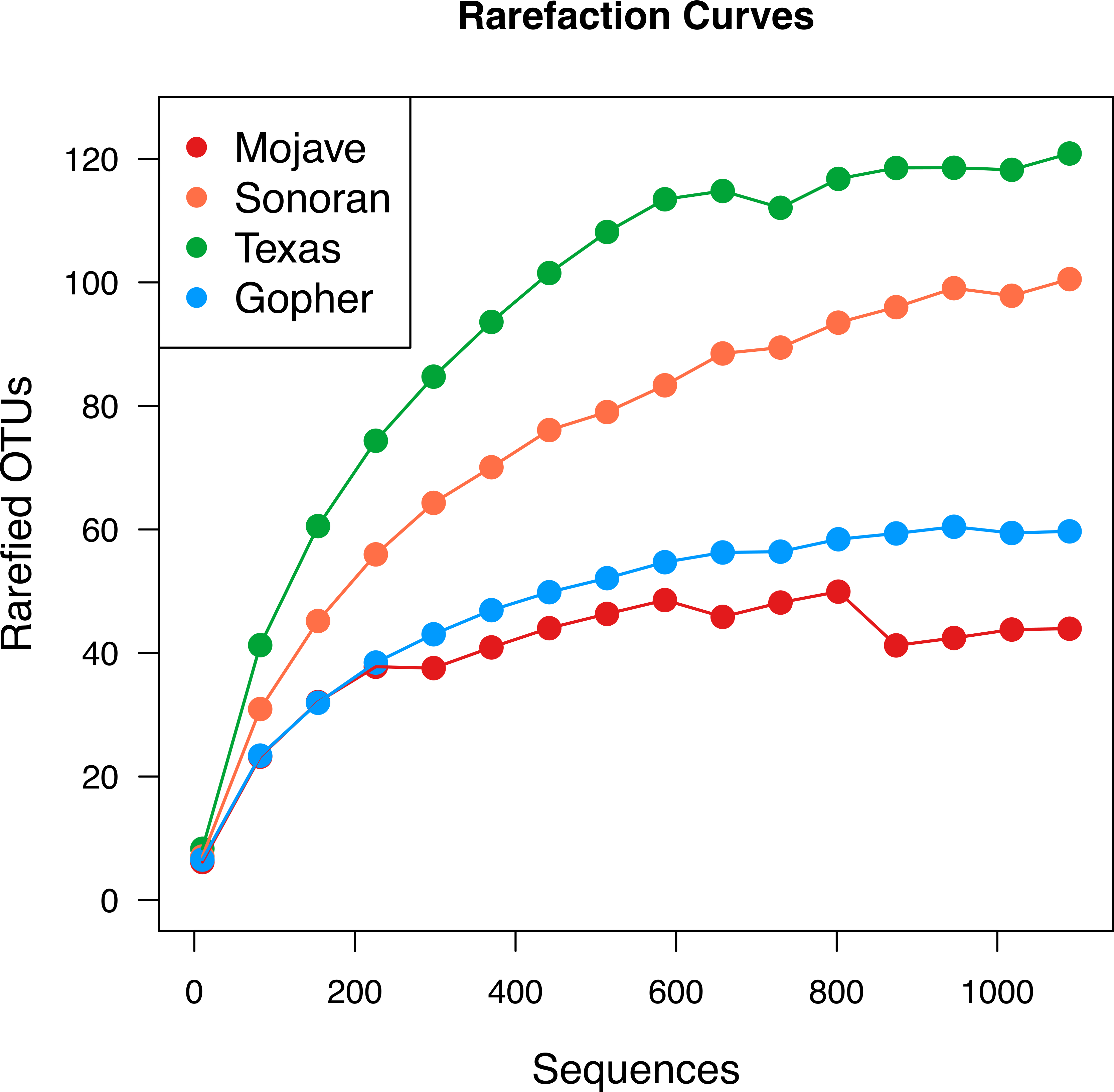


Figure S2. Rarefaction curves of OTU richness by species excluding *Chelonobacter* OTUs.


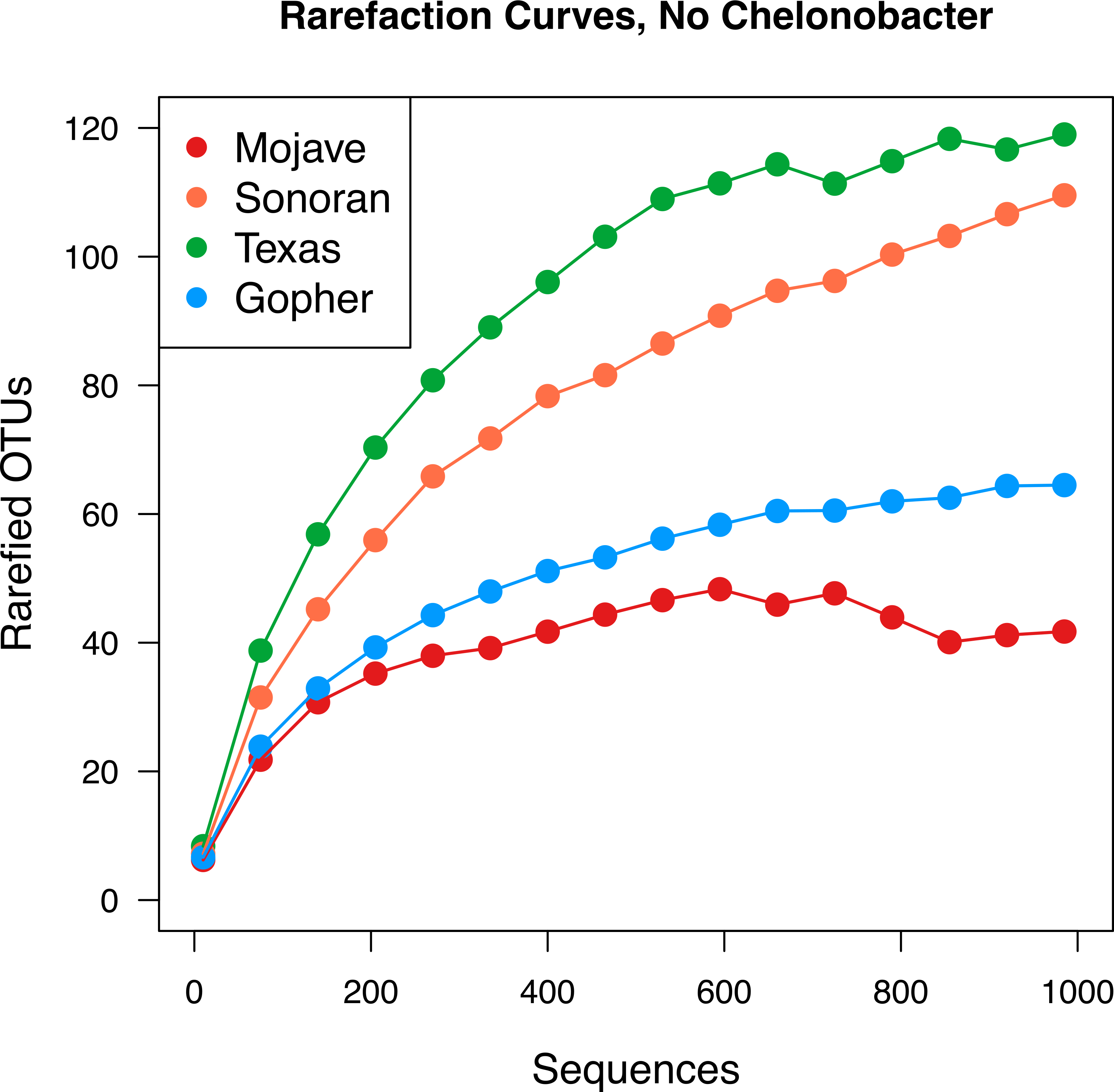


Figure S3. OTU richness in North American *Gopherus* tortoise nasal samples by geographic sampling site. Data subset to 600 sequences per sample. (a) Mojave desert tortoises, *G. agassizii*. ED = Eldorado, FV = Fenner Valley, SV = South Las Vegas, CS = Coyote Springs, RC = Red Cliffs. (b) Sonoran desert tortoises, *G. morafkai*. CB = Cave Buttes, SL = Sugarloaf, TU = Tucson. (c) Texas tortoises, *G. berlandieri*. CH = Chaparral Wildlife Management Area, WE = West Rio Grande, EA = East Rio Grande. (d) Gopher tortoises, *G. polyphemus.* PD = Perdido, RY = Rayonier, USF = University of South Florida reserve. Letters above the boxplots denote significantly different groups from Tukey’s post-hoc tests of analysis of covariance, with total sequences as a covariate. Boxplots indicate the mean, interquartile range, reasonable range of the data, and outliers.


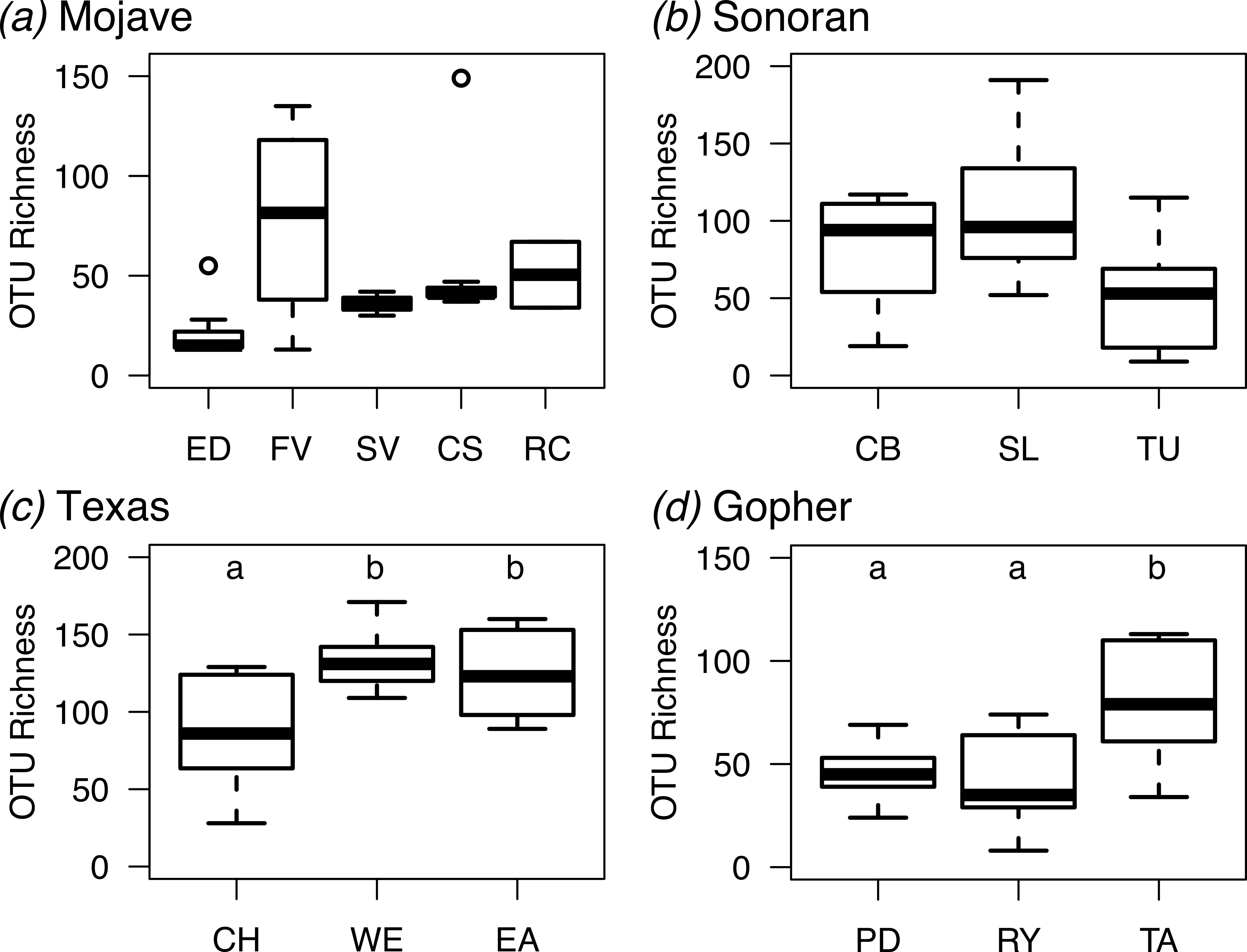


Figure S4. Core microbiome, designated as OTUs present in 50% of the samples per host species, for three North American *Gopherus* tortoise species. Core OTUs colour-coded by bacterial family.


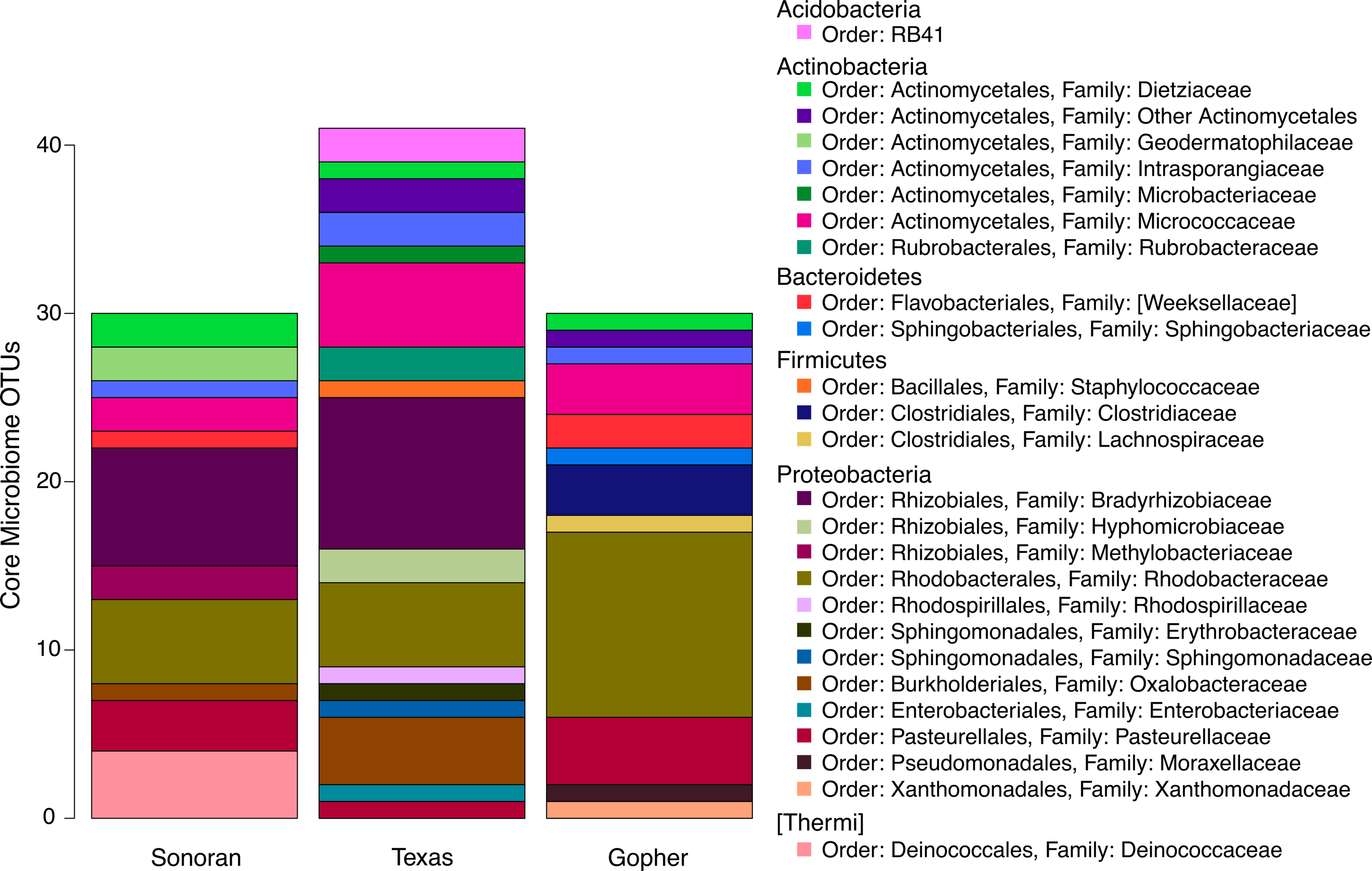


Figure S5. Core microbiome, designated as OTUs present in 75% of the samples per sampling site, for four North American *Gopherus* tortoise species. Core OTUs colour-coded by bacterial family.


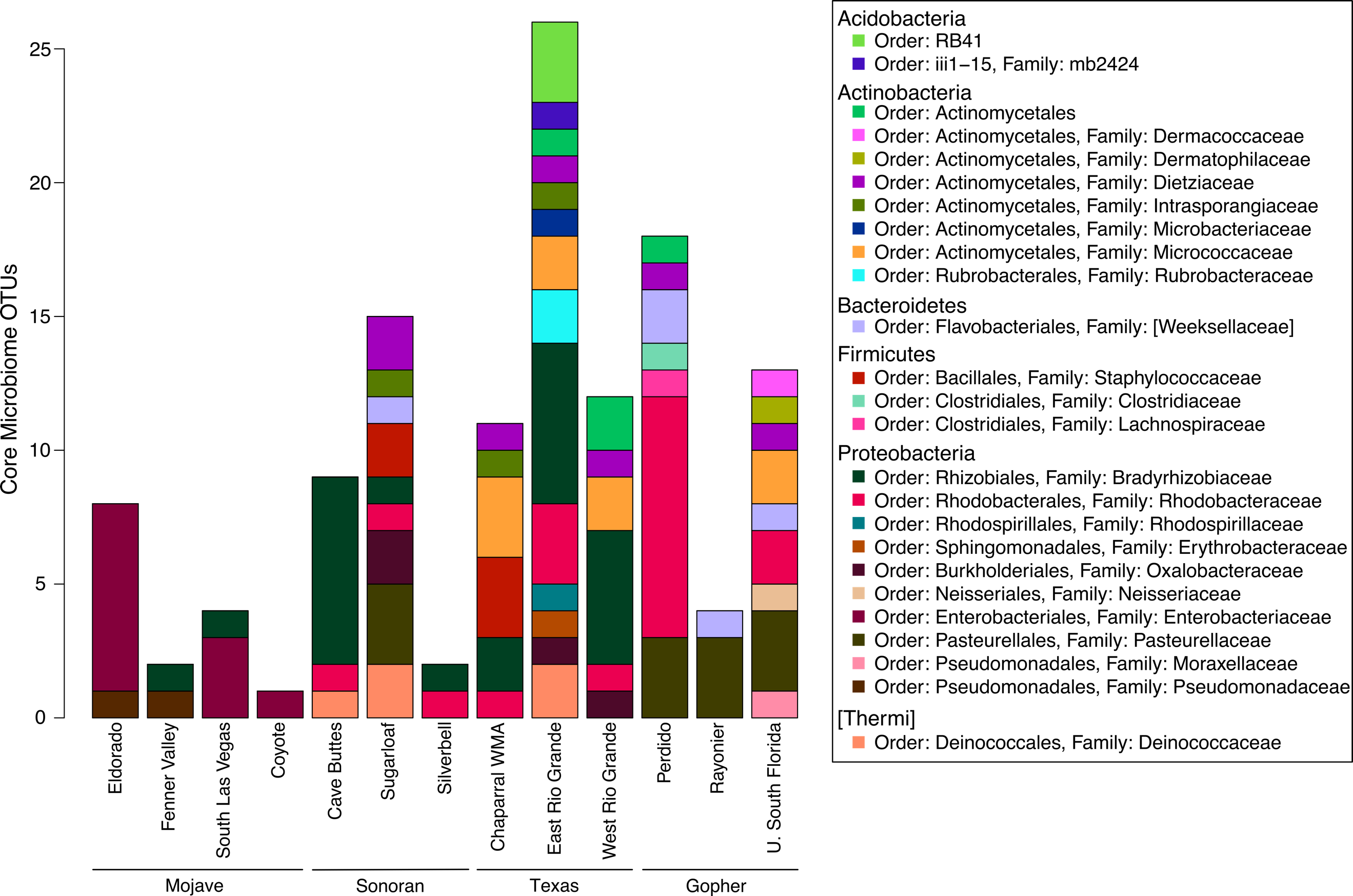

Supplement: Supplementary Figures [file rsos181068supp1.docx]
